# A Chinese herbal formula "Gan-Lu-Yin" suppresses vascular smooth muscle cell migration by inhibiting matrix metalloproteinase-2/9 through the PI3K/AKT and ERK signaling pathways

**DOI:** 10.1186/1472-6882-12-137

**Published:** 2012-08-24

**Authors:** Yi-Chung Chien, Ming-Jyh Sheu, Chieh-Hsi Wu, Wen-Hsin Lin, Ying-Yi Chen, Po-Liang Cheng, Hsu-Chen Cheng

**Affiliations:** 1 Department of Life Sciences, and Agricultural Biotechnology Center, National Chung Hsing University, 250 Kuo Kuang Road, Taichung, 402, Taiwan; 2 School of Pharmacy, China Medical University, 91 Hsueh-Shih Road, Taichung, 404, Taiwan

**Keywords:** Chinese herbs, Gan-Lu-Yin, Migration, Restenosis, Vascular smooth muscle cell

## Abstract

**Background:**

This study was to explore the effects of Gan-Lu-Yin (GLY) on the migration of vascular smooth muscle cells (VSMCs) induced by fetal bovine serum and on neointima formation in a rat model of carotid artery balloon injury.

**Methods:**

VSMCs were treated with different concentrations of GLY, and then analyzed with Flow cytometric analysis, zymography, transwell, and western blotting. SD rats received balloon-injury were analyzed with H&E staining.

**Results:**

Our results showed that GLY significantly decreased the thickness of neointima. The inhibition by non-cytoxic doses of GLY of VSMCs migration was through its negative regulatory effects on phosphorylated ERK1/2, PI3K/AKT, and FAK. The data showed that GLY can inhibit the migration of VSMCs cells, and might block injury-induced neointima hyperplasia via the inhibition of VSMCs migration, without inducing apoptosis.

**Conclusions:**

These observations provide a mechanism of GLY in attenuating cell migration, thus as a potential intervention for restenosis.

## Background

Angiographic restenosis is still a major limitation for the clinical application of percutaneous transluminal coronary angiography (PTCA). It has been well documented that restenosis is a complex and multifactorial process involving arterial remodeling and neointima hyperplasia, which might be caused by endothelial disruption, inflammation, cell proliferation and migration of vascular smooth muscle cells (VSMCs) [1]. Thereby, the pharmacological effects of drug-eluting stents for clinical trials were focused on the prevention of migration and proliferation [2].

Gan-Lu-Yin (GLY), a Chinese herbal formula, consists of *Rehmannia glutinosa*, *Liriope spicata* (Thunb.) Lour, *Eriobotrya japonica* (Thunb.) Lindl, *Citrus sinensis* Osbeck, *Glycyrrhiza uralensis* Fisch, *Artemisia capillaris* Thunb., *Dendrobium nobile* Lindl., and *Scutellaria baicalensis* Georgi. Many of the GLY components, such as *R. glutinosa*, *L. spicata* (Thunb.) Lour, *E. japonica* (Thunb.) Lindl., *C. sinensis* Osbeck, *G. uralensis* Fisch, *A. capillaris* Thunb., *D. nobile* Lindl., and *S. baicalensis* Georgi, have been used as health foods for a long history in Taiwan. Besides, GLY has been used as a popular drink to manage constipation or oral ulcers. This formula is used to expel the heat, remove the dampness, resolve inflammation, and clean the blood according to traditional Chinese medicinal prescriptions, Tai Ping Hui Min He Ji Ju Fang. Numerous studies mentioned that some of the single ingredients within the GLY formula have biological effects on antitumor potential by inhibiting cell proliferation or inflammation [3-5]. Our recent observations suggested that GLY extract has an inhibitory effect on angiogenesis, which in turn may prevent tumor growth, and its mechanism might be partially associated with blocking vascular endothelial growth factor (VEGF) protein expression of HUVEC [6]. However, the investigation regarding the attenuation of balloon injury-induced neointima formation has never been explored. In this study, our results have shown that GLY extract has an inhibitory effect on balloon injury-induced neointima formation. Moreover, the effect of GLY formula on fetal bovine serum (FBS)-induced VSMCs cells migration was studied. The present study was designed to explore the anti-proliferative and -migration effects of GLY on vascular smooth muscle cells and to evaluate its molecular mechanisms.

## Methods

### Reagents and chemicals

Dulbecco’s Modified Eagle’s Medium (DMEM), 3-(4, 5-dimethylthiazolyl-2)-2, 5-diphenyltetrazolium bromide (MTT), RNase A, and other chemicals were obtained from Sigma Chemical Co. (St. Louis, MO, USA). Trypsin - EDTA, fetal bovine serum (FBS), and penicillin/streptomycin were from Gibco Life Technologies, Inc. (Paisley, UK). Cell culture supplies were purchased from Costar (Corning, Inc., Cypress, CA, USA). The antibody against PI3K, Akt, MAPK/extracellular signal-regulated kinase (ERK) 1/2, and phosphorylated proteins were purchased from Cell Signaling Technology (Beverly, MA, USA). Anti-ERK1/2, anti-PI3K, antifocal adhesion kinase (FAK), anti-p-FAK, and horseradish peroxidase-conjugated goat antimouse IgG antibody were purchased from Santa Cruz Biotechnology Co. (Santa Cruz, CA, USA).

### Materials

The ingredients of GLY, *R. glutinosa* (rehmannia), *L. spicata* (Thunb.) Lour (lilyturf), *E. japonica* (Thunb.) Lindl. (loquat leaves), *C. sinensis* Osbeck (sweet orange), *G. uralensis* Fisch (licorice root), *A. capillaris* Thunb. (capillaris), *D. nobile* Lindl. (dendrobium), and *S. baicalensis* Georgi (baical skullcap root), were provided from the Pharmacy Department of China Medical University Hospital, Taichung, Taiwan. All other reagents were purchased from Sigma-Aldrich (St. Louis, MO, USA).

### Preparation of GLY extract

The ingredients of the GLY formula were equally weighed (about 1 kg) and soaked in 10 L of 50% ethanol solution (extractive solvent) for 3 days at room temperature. The solid residue of the above soaked herbs was filtered and discarded through a Buchner funnel lined with Whatman filter paper, and the filtrate was concentrated to paste by distillation under reduced pressure. The series concentrations (0.0625, 0.125, 0.25, 0.5, 1.0 and 2.0 mg/mL) of GLY extract were further diluted with deionized water for the subsequent studies.

### Cell culture

VSMCs derived from rat thoracic aorta were obtained from Food industry Research and Development Institute (Hsinchu, Taiwan). Cells were maintained in Dulbecco’s modified Eagle’s medium (DMEM) supplemented with 10% (v/v) fetal bovine serum (FBS), 2 mM L-glutamine, 1 mM sodium pyruvate, 100 units of penicillin, and 100 mg of streptomycin per mL. The cells were kept in a humidified 5% CO_2_/95% air incubator at 37°C.

### Cytotoxicity assay

MTT assay was performed to measure the cytotoxicity of GLY on VSMCs were seeded in ninety-six-well plates with 1 × 10^4^ cells/well in DMEM supplemented with 10%FBS. After 24 h, cells were washed with PBS and then exposed to either 10% FBS alone or serial dilutions (0.0625, 0.125, 0.25, 0.5, 1.0 and 2.0 mg/mL) of GLY. After 24 h, the number of viable cells was determined by ELISA reader (Anthos 2001; Anthos Labtec, Salzburg, Austria).

### Flow cytometric analysis

Cellular total DNA contents of the treated cells were assessed using flow cytometry following propidium iodide (PI) staining. Cells were analysed using a FACScan flow cytometer (Becton Dickinson, San Jose, CA, USA). PI fluorescence was linearly amplified and both the area and width of the fluorescence pulse were measured. Ten thousand events were acquired, and the percentages of hypodiploid (apoptotic, sub-G_1_) events and percentages of cells in the G_0_/G_1_, S and G_2_-M phases were determined using the DNA analysis software ModFitLT, version 2.0 (Verity Software, Topsham, ME, USA).

### Wound healing assay

For cell motility determination, VSMCs (3 × 10^4^) were seeded in a 6-well tissue culture plate and grown to 80 - 90% confluence. After aspiration of the medium, the center of the cell monolayers was scraped with a sterile micropipet tip to create a denuded zone (gap) of constant width. Subsequently, cellular debris was washed with PBS, and VSMCs cells were exposed to various concentrations of GLY (0, 0.0625, 0.125, 0.25, and 0.5 mg/mL). Wound closure was monitored and photographed at 0 and 18 h with a Nikon inverted microscope. To quantify the migrated cells, pictures of the initial wounded monolayers were compared with the corresponding pictures of cells at the end of the incubation. Artificial lines fitting the cutting edges were drawn on pictures of the original wounds and overlaid on the pictures of cultures after incubation. Cells that had migrated across the white lines were counted in six random fields from each triplicate treatment.

### Cell migration assay

VSMCs migration was assayed in transwell chambers (Millipore) according to the method reported by Huang et al. with some modifications [7]. Briefly, transwell chambers with 6.5 mm polycarbonate filters of 8 μm pore size were used. VSMCs cells (5 × 10^5^ mL^-1^) and 0, 0.0625, 0.125, 0.25, and 0.5 mg/mL of GLY were suspended in DMEM (100 μL, serum free), placed in the upper transwell chamber, and incubated for 18 h at 37°C. Then, the cells on the upper surface of the filter were completely wiped away with a cotton swab, and the lower surface of the filter was fixed in methanol, stained with Giemsa, and counted under a microscope at a magnification of 100× and 200×. For each replicate, the VSMCs cells in 10 randomly selected fields were determined and the counts were averaged.

### Determination of MMP-2 and MMP-9 by zymography

MMP in the medium released from VSMCs was assayed using gelatin zymography (7.5% zymogram gelatin gels) according to the methods reported by Huang et al., with some modification [7]. Briefly, the culture medium was electrophoresed (120 V for 90 min) in a 7.5% SDS-PAGE gel containing 0.1% gelatin. The gel was then washed at room temperature in a solution containing 2.5% (v/v) Triton X-100 with two changes and subsequently transferred to a reaction buffer for enzymatic reaction containing 1% NaN_3_, 10 mM CaCl_2_ and 40 mM Tris–HCl, pH 8.0, at 37°C with shaking overnight (for 12-15 h). Finally, the eletrophoretic gel was stained for 30 min with 0.25% (w/v) coomassie blue in 10% acetic acid (v/v) and 20% methanol (v/v) and destained in 10% acetic acid (v/v) and 20% methanol (v/v).

### Western blotting analysis

VSMCs cultured in six-well plates were incubated with GLY at 0.125, 0.25 and 0.5 μg/mL in DMEM containing 10% FBS for 24 h. The cells were then lysed in a buffer containing 2% SDS, 50 mm-dithiothreitol, 62.5 mM -Tris–HCl, pH 6.8, followed by incubation at 95°C for 5 min. Samples were separated using SDS-PAGE, transferred to polyvinylidene fluoride (PVDF) membranes, blocked with 5% non-fat dry milk in PBS-Tween for 1 h, and then probed with the desired antibodies (anti-phosphorylated focal adhesion kinase (p-FAK; 1:1000), anti-phosphorylated ERK1/2 (1:1000), anti PI3K (1:2000), anti-AKT (1: 200), anti-pAKT (1:200), anti-MMP-2 (1:2000), anti-MMP-9 (1:1000), anti-TIMP-1 (1:1000)and anti-TIMP-2 (1:200); Novus Biologicals, Littleton, CO, USA) overnight at 4°C. The blots were then incubated with horseradish peroxidase-linked secondary antibody for 1 h followed by development with the electrochemical luminescence (ECL) reagent and exposure to Hyperfilm (Amersham, Arlington Heights, IL, USA).

### Balloon angioplasty

Sprague–Dawley rats weighing 350-400 g were purchased from National Science Council (Taipei, Taiwan). Sixteen rats were used and divided as two groups including total injury control without GLY (n = 8), and 0.5 g/mL (n = 8) of GLY-treated groups. Animals were housed in a 12 h light–dark cycle with free access to food and water. All experimental procedures involving animals were approved by the ethics committee of the Institutional Animal Care and Use Committee of China Medical University. The rats were anaesthetised with 3.6% (w/v) chlorohydrate (1 mL/100 g, intraperitoneally). Angioplasty of the carotid artery was performed using a balloon embolectomy catheter as described previously [8].

### Statistical analysis

Values are expressed as means ± SD and analyzed using one-way ANOVA followed by LSD test for comparisons of group means. All statistical analyses were performed using SPSS for Windows, version 10 (SPSS, Inc.). The level of significance was set at *P* value <0.05.

## Results

### GLY inhibited FBS-induced VSMCs proliferation

**Figure 1:**
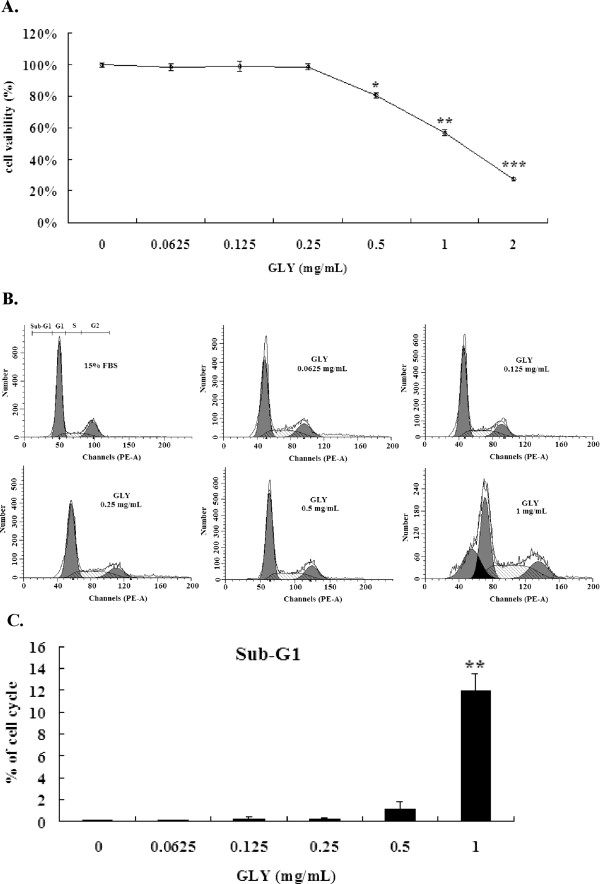
** Effects of GLY on cell viability and cell cycle analysis.** (**A**). Effects of GLY on cell growth of VSMCs by MTT assay. The cells were incubated for 24 h with 10% fetal bovine serum alone (control) or with different concentrations of GLY (0.0625, 0.125, 0.25, 0.5, 1 and 2 mg/mL). (**B**). Flow cytometric analysis of GLY on the cell cycle of VSMCs cells. All the cells were treated with 15% fetal bovine serum with the addition of GLY at 0.0625,0.125,0.25,0.5,1 mg/mL for 18 h. (**C**). The percentage of subG1 under flow cytometric analysis. Values are means of three separate experiments, with standard errors represented by vertical bars. Mean value was significantly different from that of the control group :* *p* < 0.05,** *p* < 0.01, *** *p* < 0.001.

Since outgrowth of VSMCs has been regarded as the major factor leading to restenosis, we performed the MTT assay to determine the inhibitory effects of GLY on cell viability of VSMCs. As shown in Figure 1A, GLY inhibited VSMCs viability in a concentration-dependent manner. The inhibitory effect of GLY on cell viability became significant at 0.5 mg/mL (*P* < 0.05), 1 mg/mL (*P* < 0.01) and 2 mg/mL (*P* < 0.001) after 24 h incubation.

### Effects of GLY on cell cycle

Since the MTT showed that GLY at 0.5, 1 and 2 mg/mL significantly suppressed cell viability, we postulated that the inhibitory effects of GLY on cell viability might be mediated by apoptosis. We chose GLY at 0.0625, 0.125, 0.25, 0.5 and 1.0 mg/mL to determine its effects on cell cycle and apoptosis. The results demonstrate that treatment for 24 with GLY at 0.0625, 0.125, 0.25 and 0.5 mg/mL had no effects on the cells apoptotic in the sub-G_1_ phase, however, treatment for 24 h. with GLY at 1.0 mg/mL demonstrated significant apoptosis (Figures 1B and 1C).

### Non-cytotoxic doses of GLY inhibited FBS-stimulated VSMCs motility in wound healing assay

**Figure 2:**
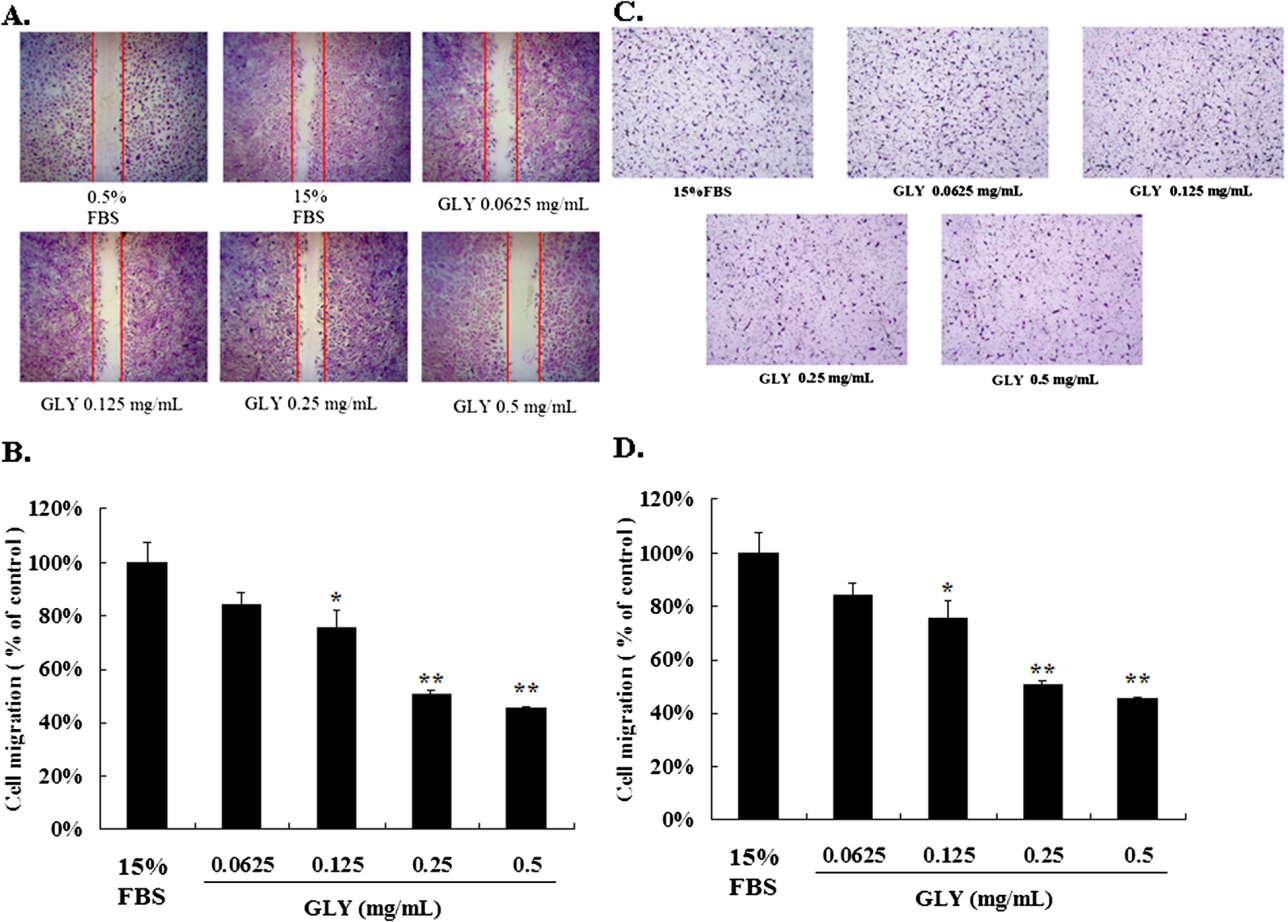
** Effects of GLY on wound healing migration of VSMCs cells.** Wound was introduced by scraping confluent cell layers with a pipet tip. VSMCs cells were incubated with GLY (0.0625, 0.125, 0.25 and 0.5 mg/mL) for 18 h, and the migration distances of cells were calculated. (**A**) Representative photographs of invading cells that received either control (15% FBS) or GLY treatment. (**B**) Migrated cells across the black lines were counted in six random fields from each treatment. The mean number of cells in the denuded zone is quantified by three independent experiments. (**C**). Effects of GLY on transwell migration assay vascular smooth muscle cells. VSMCs were incubated with GLY (0.0625, 0.125, 0.25 and 0.5 mg/mL) for 18 h, and (**D**). The transwell migration cells were calculated. Photos of the migration VSMCs cells were taken under a microscope (100-fold magnification). Mean value was significantly different from that of the control group (15% FBS): * *p* < 0.05,** *p* < 0.01.

We assessed the effect of GLY on the migration of VSMCS cells using the wound healing assay in which the confluent monolayer was scraped with a sterile micropipet tip to create a scratch wound. As shown in our data, GLY at 0.25 and 0.5 mg/mL inhibited the migration of VSMCS cells (Figures 2 A and 2B).

### Non-cytotoxic doses of GLY inhibited FBS-stimulated VSMCs migration

The transwell assay was used to investigate the migration of VSMCs cells at 18 h after GLY treatment. We found that GLY added at 0.125, 0.25 and 0.5 mg/mL significantly decreased the migration (Figures 2 C and 2D) of VSMCs cells.

### Non-cytotoxic doses of GLY inhibits the Release of MMP-2 and MMP-9 in VSMCs Cells

**Figure 3:**
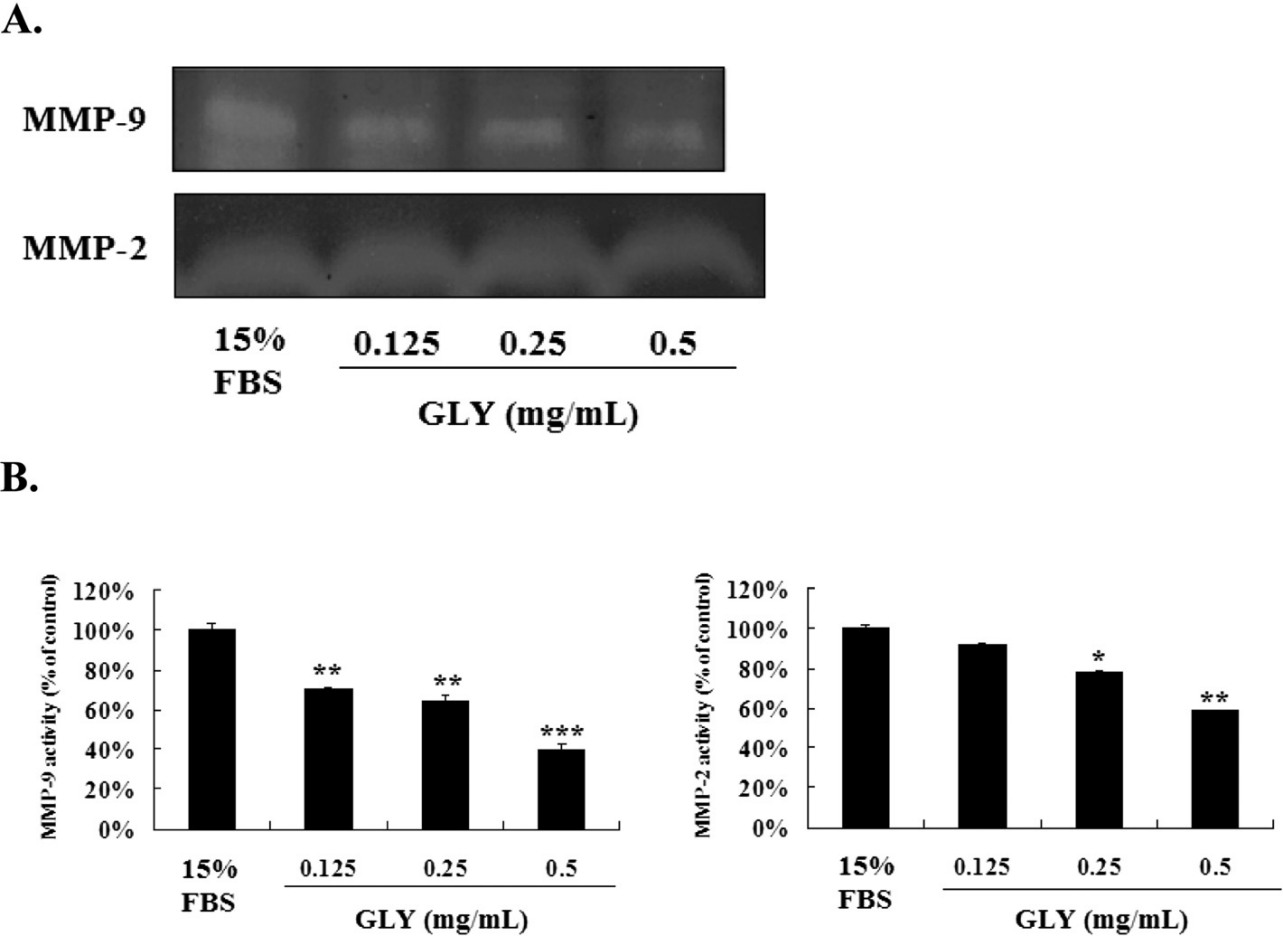
** GLY inhibits the release of MMP-2 and MMP-9 in VSMCs.** (**A**). To examine the possible antimigration mechanisms of GLY, we determined the activity of MMP-2 and MMP-9 in culture media of VSMCs cells by zymographic analysis. (**B**). GLY inhibited MMP-9 and MMP-2 activities in a concentration-dependent manner. Values are means of three separate experiments, with standard errors represented by vertical bars. Mean value was significantly different from that of the control group :* *p* < 0.05,** *p* < 0.01, *** *p* < 0.001.

To examine the possible antimetastatic mechanisms of GLY, we determined the activity of MMP-2 and MMP-9 in culture media of VSMCs cells by zymographic analysis. In the absence of treatment, VSMCs cells constitutively secreted both MMP-9 and MMP-2. As shown in Figure 3A, GLY inhibited MMP-9 and MMP-2 activities in a concentration-dependent manner after incubation for 18 h (Figures 3 B and 3C).

### Non-cytotoxic doses of GLY activated TIMPs, MMP-2, and MMP-9 expressions in VSMCs cells

**Figure 4:**
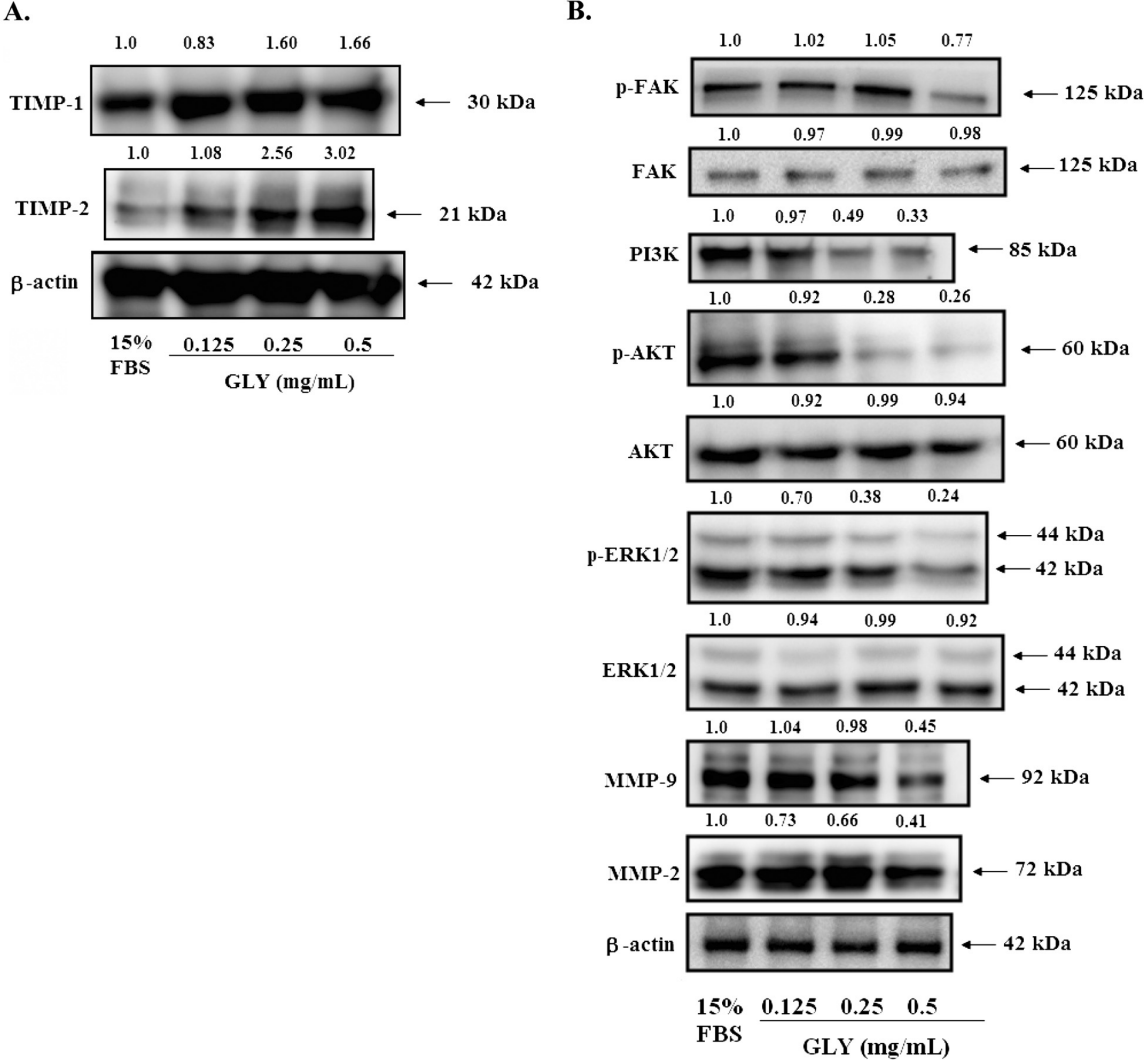
** Molecular mechanisms of GLY on FBS-induced VSMCs.** (**A**). Effects of GLY on TIMP-1, TIMP-2, MMP-2, MMP-9 proteins expression. VSMCs were treated with 0.125, 0.25 and 0.5 mg/mL for 24 h, and cell lysates were subjected to SDS-PAGE followed by Western blotting. (**B**). Dose-dependent effects of GLY on the protein expression level of FAK, the phosphorylated FAK, ERK1/2, the phosphorylated ERK1/2, PI3K, MMP-2, MMP-9, AKT and the phosphorylated AKT. VSMCs were treated with 0.125, 0.25 and 0.5 mg/mL of GLY for 24 h. The expression of these proteins were analyzed by Western blotting. β-actin was used as a loading control.

To further explore the modulation of pro-MMP and MMP activation by GLY, we determined TIMP-1/2 and MMP2/9 proteins expression levels. As shown in Figure 4A, GLY strongly increased TIMP-1/2 activity and also decreased MMP-2/9 activity in a concentration-dependent manner.

### Non-cytotoxic doses of GLY inhibited phosphorylated focal adhesion kinase and phosphorylated extracellular signal-regulated kinase

To evaluate the effect of GLY on FAK and ERK1/2 protein expression, VSMCs was treated with GLY at 0.125, 0.25 and 0.5 mg/mL for 30 min. As shown in Figure 4B, GLY had no effect on FAK expression. In addition, GLY at 0.25 and 0.5 mg/mL significantly suppressed the phosphorylation of FAK (p-FAK) in VSMCs cells. (Figure 4B). We analyzed the phosphorylation of ERK1/2 in VSMCS cells after treatment with GLY (0.125, 0.25 and 0.5 mg/mL) for 30 min. Data in Figure 4B showed that GLY significantly affect phosphorylated ERK1/2 at concentrations used (0.25 and 0.5 mg/mL).

### Non-cytotoxic doses of GLY inhibited the PI3K/Akt signaling in VSMCs cells

To further investigate the involvement of PI3K/Akt, a series of experiments was performed to measure the expression of candidate signaling molecules upon GLY stimulation. The results showed that incubation of VSMCs cells with GLY (0.125, 0.25 and 0.5 mg/mL) led to a concentration-dependent decrease of PI3K and p-Akt levels (Figure 4B).

### Extract of GLY inhibited balloon injury-induced neointima formation on the carotid artery

**Figure 5:**
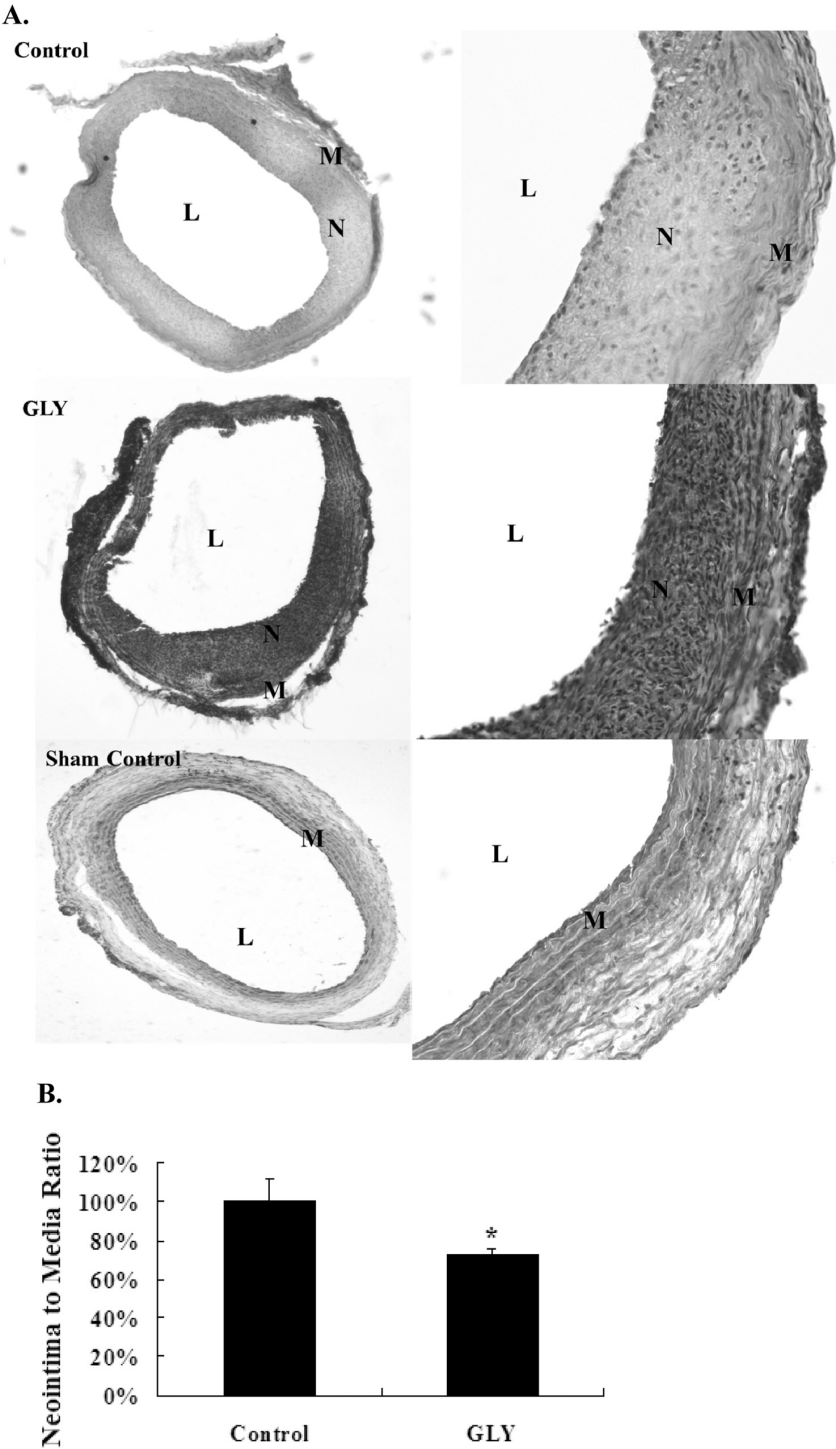
** Responses of rat carotid arteries to balloon injury, and the effects of extract of GLY on balloon injury.** (**A**). The left panel represents the low-power (100×) observations from a balloon-injured vessel, a balloon-injured vessel treated with GLY at 0.5 g/ml,and uninjruied vessel (sham control). And the right panel represents the high-power (200×). (L, lumen; N, neointima; M, medium.). (**B**). The control group shows a significantly higher neointima to media ratio as compared with the groups treated with extract of GLY at 0.5 g/mL. Values are means of two separate experiments, with standard errors represented by vertical bars. Mean value was significantly different from that of the control group:* *p* < 0.05.

To test the efficacy of GLY in inhibiting neointima formation, Sprague–Dawley rats were applied with different concentrations of GLY (0.5 g/mL) for 14 days following balloon injury. After 2 weeks of balloon injury, the injured arteries were harvested and subjected to histological analysis for neointima formation assay. Intimal hyperplasia induced by balloon injury was evident as compared with the normal control (Figure 5A). The present results showed that GLY (0.5 g/mL) were effective in preventing neointima formation (Figure 5B). However, GLY at 0.125 g/mL and 0.25 g/mL did not show any influence on balloon injury-induced neointimal formation (data not shown). Using computerized image analysis, we calculated the area ratio of intimal and media layers; we found a reduction of 24% in the area ratio of GLY-treated groups as compared with the balloon-injured control group by GLY at 0.5 g/mL of GLY, respectively (Figure 5B).

## Discussion

Earlier studies have shown that pretreatment with antioxidants can significantly reduce balloon injury-induced neointima formation [9]. Our recent data have demonstrated that the MDA levels were significantly reduced by GLY treatment. Also, GLY extracts are effective in scavenging α,α-diphenyl-β-pricrylhydrazyl (DPPH) radicals (data not shown). We found GLY extract could induce apoptosis of vascular endothelial cells in a dose-dependent manner [6]. GLY having antioxidation and free radical scavenging activities may be developed as another potential candidate to prevent restenosis. To test whether GLY can be an effective therapeutic intervention for balloon injury, administration of GLY in preventing abnormal cell proliferation were evaluated at various concentrations in in vivo. And, we aimed to investigate if GLY contributed to the attenuation of VSMCs migration and therefore, explored its molecular mechanisms. The present study provides a general insight into the molecular mechanism of GLY in preventing the outgrowth of smooth muscle cells, which is a potential intervention for balloon injury-induced neointima formation.

Recently, Lin et al. mentioned that at least 14 main components of GLY have been identified using liquid chromatography-mass spectrometry (LC-MS) and inductively coupled plasma MS (ICP-MS), and these components included baicalin, baicalein, oroxylin A-7-O-glucuronide, wogonin-7-Oglucuronide, wogonin, and oroxylin A in *Radix Scutellariae*; naringin and neohesperidin in *Aurantii fructus*; and liquiritigenin, liquiritin, and glycyrrhizic acid in *Radix Glycyrrhizae*[10]. In our previous study, the indicator components of GLY extract such as baicalein, chlorogenic acid, and glycyrrhizic acid have been detected by HPLC/ESI-MS analysis [6]. Four other components such as baicalin, naringin, naringenin, and vanillin have also been detected from our study (data not shown). It has been mentioned that baicalin and baicalein, two flavonoid compounds, have inhibitory effects on endothelial cell proliferation, migration, and differentiation [11,12]. Endothelial cell proliferation, adhesion, and migration are early essential events for mediating angiogenesis. Hsieh et al. indicated that baicalein significantly up-regulated protein expression of integrins and vinculin in rat endothelial cells, which will result in the increase of focal contact formation and cell adhesion to fibronectin and vitronectin [11]. Coordination and balance between cell adhesion to and detachment from extracellular matrix (ECM) is crucial for cell motility, so unilateral increasing cell adhesion will severely affect endothelial migration and angiogenesis processes. In addition, it has been reported that baicalein and baicalin could inhibit cell proliferation of tumor cells and induce apoptosis of myeloma cells [13,14]. Sun et al. found that oroxylin A suppresses invasion through inhibiting cell migration and down-regulating the expression of matrix metalloproteinase-2/9 in human breast cancer cells [15]. Wogonin has been evidenced to inhibit inflammatory cytokine-induced angiogenesis of HUVEC and suppress the VEGF-stimulated migration and tube formation of HUVEC [16,17]. Wogonin effectively suppresses TNF-α-induced HASMC migration through the selective inhibition of MMP-9 expression [18]. Schindler et al. reported that naringin could decrease tumorinduced vascular proliferation by reducing the release of VEGF from human tumor cells [19]. The inhibitory effect of liquiritigenin on tube formation of vascular endothelial cells has also been reported [20]. However, glycyrrhizic acid, the main component of licorice, has been investigated for its ability to increase tube formation [20]. Jung et al. mentioned that caffeic acid and its derivative, CAPDE ([3-(3,4-dihydroxy-phenyl)-acrylic acid 2-(3,4-dihydroxy-phenyl)-ethyl ester]) can suppress tumor angiogenesis by blocking VEGF expression in human renal carcinoma cells [21]. Although chlorogenic acid is also a caffeic acid derivative compound, so far there has not been direct evidence to point out the association with the effect on antiangiogenesis. However, previous literature indicated that it is a new type of strong matrix metalloproteinase-9 inhibitor, which might prevent the invasion and metastasis of malignant cancer cells [22]. According to the above statements, we suppose that these ingredients from GLY might contribute the inhibition of proliferation and migration in VSMCs.

VSMCs proliferation and migration are important contributors to neointima formation after balloon injury. Therefore, modulation of VSMCS growth has critical therapeutic implications [23]. In the present study, for the first time we show that GLY attenuates neointima hyperplasia after balloon angioplasty. To evaluate if GLY was effective in suppressing neointima formation following balloon angioplasty, an in vivo study using rat carotid artery as a model was conducted in the present study. Sixteen rats were used and divided as two groups including total injury control without GLY, and 0.5 g/mL of GLY-treated groups. A balloon catheter was first surgically inserted into rat carotid arteries to induce injury. At 2 weeks after balloon injury, the arteries were subjected to histological analysis and GLY was found to significantly reduce neointima formation 14d following arterial injury (Figure 5A). The neointima:media ratios of arterial samples from animals treated with GLY were significantly lower than those of the total injury control tissues (Figure 5B).

GLY was carried out on its mechanisms on serum-induced VSMCs migration behavior. VSMCs migration is important contributors to neointima formation after balloon injury. We first demonstrated that GLY exerted potent inhibitory effects on the growth of VSMCs (Figure 1A). Since the MTT showed that GLY at 1 and 2 mg/mL significantly suppressed cell viability, we postulated that the inhibitory effects of GLY on cell viability might be mediated by apoptosis. We chose GLY at 0.0625, 0.125, 0.25, 0.5 and 1.0 mg/mL to determine its effects on cell cycle and apoptosis. The results demonstrate that treatment for 24 with GLY at 0.0625, 0.125, 0.25 and 0.5 mg/mL had no effects on the cells apoptotic in the sub-G_1_ phase (Figures 1 B and 1C). Therefore, GLY at 0.125, 0.25 and 0.5 mg/mL was conducted on its role in migration analysis. We found that GLY has unique biological properties; it does not affect cell survival (Figure 1A) and apoptosis (Figures 1 B and 1C) at concentrations up to 0.5 mg/mL, whereas it has profound effects on migration at 0.125, 0.25 and 0.5 mg/mL (Figures 2 and 3).

The MAPK pathways play an important role in promoting VSMCs proliferation [24]. The MAPK signaling pathway was activated by vascular injury and the inhibition of activated ERK pathway by drugs or gene therapy can reduce neointima hyperplasia [25]. Our previous reports show that the *ras* gene is involved in the underlying mechanisms for neointima formation by balloon injury [26]. Therefore, several proteins involved in these pathways were examined. FAK is a protein involved in transducing extracellular growth signal from matrix via integrin interaction. Down-regulation of FAK may result in cell-cycle arrest [27]. Activated FAK in cancer cells relays signals through multiple downstream targets. For example, activated FAK binds the Src-homology domain 2 (SH2) of PI3K, thereby transporting the catalytic subunit of PI3K to the membrane, where it catalyzes the phosphorylation of inositol lipids [28]. The residues surrounding Tyr^397^ can also constitute a sequence that binds to the Ras signaling pathway. The downstream targets of the Ras signaling pathway include ERK1/2 [29]. Indeed, these pathways are activated during integrin binding to the ECM, resulting in the transduction of external stimuli from the ECM to the nucleus [30]. Raf, an important protein in the MAPK pathway, is responsible for signal transduction from Ras to ERK. Along the pathway, signaling of phosphorylated-ERK1/2 is also an essential element for cell proliferation. Therefore, the protein expression levels of FAK and ERK were all evaluated in the present study to explore the mode of preventive action of GLY against neointima formation by balloon injury. In this study, we found that GLY inhibited the activation of FAK, as evidenced by reduced phosphorylation of FAK (Figure 4B). We also demonstrated in the present study that the level of phosphorylated-ERK was down-regulated by GLY and was in accordance with the well-known function of ERK as a critical signaling molecule leading to cell proliferation and survival (Figure 4B) [31].

Phosphoinositide 3-kinase (PI3K)/AKT signaling pathway plays a major role in the regulation of cellular growth, apoptosis, and metabolism [32]. The PI3K/AKT signaling is reportedly required for VSMCs migration and proliferation, absence of AKT impairs VSMCs proliferation and migration [33]. Level of AKT kinase phosphorylation was increased by serum in our study and GLY inhibited serum-induced phosphorylation of AKT and PI3K, indicating that AKT protein is another potential target for GLY (Figure 4B). AKT mediates cell survival and growth signals by phosphorylating and inactivating pro-apoptotic proteins [34]. Therefore, these results indicate that the ERK1/2-MAPK pathway likely synergizes with other pro-proliferative signaling pathway(s), quite possibly the PI3K/AKT pathway, to regulate serum-induced VSMCs cell proliferation and migration.

The present study demonstrated that balloon injury-induced neointima formation could be markedly reduced by GLY. Its pharmacological mechanism may be associated with the down-regulation of FAK and ERK1/2 phosphorylation proteins levels. Also, downregulation of PI3K and AKT phosphorylation could be involved in the progress of the progress of VSMCs metastasis. The present results detailed the molecular mechanisms of GLY in preventing the smooth muscle cell proliferation in vitro. In the in vivo study, we found that treatments with GLY at 0.5 g/mL significantly reduced the neointima formation in rat carotid arteries after balloon injury. Our findings regarding the inhibitory effects of GLY on smooth muscle cells may shed light onto the conjunctive roles of GLY with some other pharmacological agents in preventing restenosis. These results demonstrate that GLY suppresses the migration of VSMCs via inhibiting MAPK and PI3K/AKT signaling pathways. We proposed that GLY might be a potential agent that may prove clinically useful in patients undergoing PTCA or stenting. A further study on larger animal models or even a clinical evaluation needs to be conducted to confirm the proposed approach in this aspect.

## Conclusion

We have demonstrated that GLY inhibits the migration of VSMCs cells. Mechanistically, GLY may occur through inactivation of the ERK1/2 signaling pathway, exerting inhibitory effects on FAK and pFAK protein expressions and inhibiting PI3K, and phospho-AKT levels, thereby decreasing the activities of MMP-2 and MMP-9 leading to inhibition of metastasis of VSMCs (Figure 4B).

## Abbreviations

GLY: Gan-Lu-Yin; FBS: Fetal bovine serum; VSMCs: Vascular smooth muscle cells; MTT: 3-(4,5-dimethylthiazol-2-yl)-2,5-diphenyltetrazolium bromide; ERK: Extracellular signal-regulated kinase; p-FAK: Phosphorylated focal adhesion kinase; PI: Propidium iodide; PTCA: Percutaneous transluminal coronary angioplasty.

## Competing interests

There are no conflicts of interest.

## Authors’ contribution

YCC prepared ethanol extract of GLY and animal study. MJS, CHW, WHL, YYC, and PLC performed molecular and cell biology studies of VSMCs. YCC, MJS and HCC conceived the ideas, designed the experiments and interpreted the experimental data. All authors contributed to the manuscript preparations and approved the final manuscript.

## Pre-publication history

The pre-publication history for this paper can be accessed here:

http://www.biomedcentral.com/1472-6882/12/137/prepub
